# Implementation fidelity to HIV assisted partner services (aPS) during scale-up in western Kenya: a convergent mixed methods study

**DOI:** 10.1186/s12913-023-09541-1

**Published:** 2023-05-19

**Authors:** Beatrice Wamuti, Mercy Owuor, Wenjia Liu, David Katz, Harison Lagat, George Otieno, Edward Kariithi, Paul Macharia, Sarah Masyuko, Mary Mugambi, Carey Farquhar, Bryan Weiner

**Affiliations:** 1 Department of Global Health and Population, Harvard University, Boston, USA; 2 Independent Researcher, Nairobi, Kenya; 3 School of Nursing, University of Washington, Seattle, USA; 4 Department of Global Health, University of Washington, Seattle, USA; 5 PATH- Kenya, Kisumu, Kenya; 6 Ministry of Health, Nairobi, Kenya; 7 Department of Epidemiology, University of Washington, Seattle, USA; 8 Department of Medicine, University of Washington, Seattle, USA

**Keywords:** Implementation fidelity, HIV testing, Assisted partner services, Contact tracing, Convergent mixed methods

## Abstract

**Background:**

HIV assisted partner services (aPS) is an intervention to improve HIV status awareness among sex and drug-injecting partners of people newly diagnosed with HIV (index clients). Implementation fidelity—the degree to which an intervention is conducted as intended – is critical to effectiveness, but there are limited data about aPS fidelity when delivered by HIV testing service (HTS) providers. We explored factors affecting implementation fidelity to aPS in two high-HIV prevalence counties in western Kenya.

**Methods:**

We used convergent mixed methods adapting the conceptual framework for implementation fidelity within the aPS scale-up project. This was an implementation study examining scale-up of APS within HTS programs in Kisumu and Homa Bay counties that recruited male sex partners (MSPs) of female index clients. We defined implementation fidelity as the extent to which HTS providers followed the protocol for phone and in-person participant tracing at six expected tracing attempts. Quantitative data were collected from tracing reports in 31 facilities between November 2018 and December 2020, and in-depth interviews (IDIs) were conducted with HTS providers. Descriptive statistics were used to describe tracing attempts. IDIs were analyzed using thematic content analysis.

**Results:**

Overall, 3017 MSPs were mentioned of whom 98% (2969/3017) were traced, with most tracing attempts being successful (2831/2969, 95%). Fourteen HTS providers participated in the IDIs—mostly females (10/14, 71%) with a median age of 35 years (range 25–52), who all had post-secondary education (14/14, 100%). The proportion of tracing attempts occurring by phone ranged from 47 to 66%, with the highest proportion occurring on the first attempt and lowest on the sixth attempt. Contextual factors either enhanced or impeded implementation fidelity to aPS. Positive provider attitudes towards aPS and conducive work environment factors promoted implementation fidelity, while negative MSP responses and challenging tracing conditions impeded it.

**Conclusion:**

Interactions at the individual (provider), interpersonal (client—provider), and health systems (facility) levels affected implementation fidelity to aPS. As policymakers prioritize strategies to reduce new HIV infections, our findings highlight the importance of conducting fidelity assessments to better anticipate and mitigate the impact of contextual factors during the scale-up of interventions.

**Supplementary Information:**

The online version contains supplementary material available at 10.1186/s12913-023-09541-1.

## Contributions to the literature

- In 2016, Kenya published its first HIV assisted partner services (aPS) guidelines setting the stage for the scale-up of the intervention across the country. However, there is limited data on implementation fidelity to aPS tracing protocols.
- This study shows highly successful partner tracing through aPS. However, contextual factors at the individual (provider), interpersonal (client—provider), health systems (facility) level had both positive and negative impacts on implementation fidelity.
- This highlights the importance of conducting implementation fidelity assessments during the scale-up of interventions. This will enable policymakers better anticipate and mitigate the impact of contextual factors.

## Background

Complex health interventions require a high degree of implementation fidelity, defined as the degree to which an intervention is implemented as intended by the program designers, to be effective [1]. Health interventions, described by the World Health Organization (WHO) as any activity performed with the aim of assessing, improving, promoting, and maintaining good health, often have core components that require implementation fidelity to achieve the intended results [2]. Although achieving high fidelity within controlled research settings might be relatively easy due to stringent monitoring and evaluation, this can be quite challenging in real-world settings [3]. Numerous contextual factors outside the control of practitioners such as limited resources to support hiring, training, adoption, and scale-up, jeopardize implementation fidelity as well as intended outcomes [3, 4].

Globally, there are approximately 38.4 million people living with HIV (PLWH) with an estimated 5.9 million (15%) unaware of their HIV-positive status [5]. HIV assisted partner services (aPS), where sex and drug-injecting partners of newly diagnosed HIV-positive individuals are traced, notified, tested for HIV, and linked to care if HIV-positive, was recommended by the WHO in 2016 as a strategy to improve HIV awareness [6]. This was after clinical trials, one of which was conducted in Kenya, found that the intervention was safe, effective, and cost-effective [7–10]. Consequently, the Kenyan Ministry of Health was among the first African countries to scale up aPS under its national HIV testing services (HTS) program [11]. However, there is paucity of data evaluating implementation fidelity to aPS within pragmatic settings.

Understanding and addressing contextual factors that impact the scale-up of aPS will help policymakers and implementers effectively plan their resources to ensure high quality service delivery. The WHO recommends several approaches to improve the potential success of aPS implementation [6]. First, client preferences should take precedence when selecting the aPS approach. These approaches could either be provider referral – where the HTS provider traces the sex partner without the intervention of the index client, contract referral – where the HTS provider contacts the sex partners after a given period of time if the index client is not able to, or dual referral – where the HTS provider supports the index client in disclosing their HIV status to their sex Partner(s). Second, HTS providers offering aPS should always minimize the risk of potential harm to clients by screening for intimate partner violence, offering counselling, and referring to the necessary support services [12]. Finally, providers offering aPS should ensure the highest levels of privacy and confidentiality especially in settings where HIV is stigmatized [13]. Sex partners to PLWH residing in such settings are at an increased risk of perceived (stereotyping), enacted (overt actions), or internalized (personal value) stigma [14]. HTS providers will, therefore, need to adhere to privacy agreements with their clients as not to disclose their HIV status without consent, and ensure confidentiality of data collected from participants. Such factors play a major role in implementation fidelity to aPS and ultimately in the attainment of its intended outcomes.

## Methods

### Study design

**Fig. 1:**
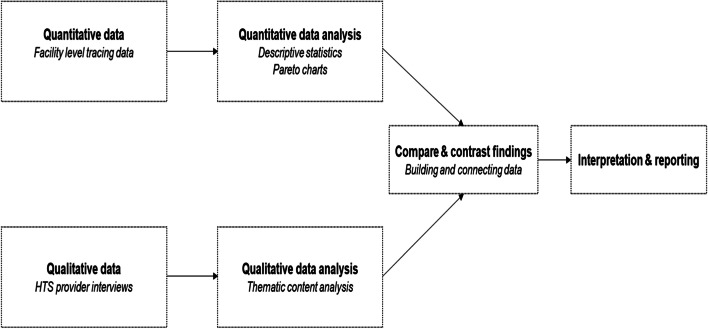
Convergent mixed methods approach

As Kenya scales up aPS in pragmatic settings, there is need to better understand the implementation fidelity to the intervention as designed. We, therefore, explored factors affecting implementation fidelity to aPS using a convergent mixed methods approach which allowed for concurrent systematic collection, analysis, and presentation of both quantitative and qualitative data (Fig. 1) [15].

We assessed compliance to tracing attempts as outlined in our study’s aPS protocol—where tracing was defined as any attempted contact with male sex partners either on phone or in-person (physical) by a HTS provider to notify them of potential HIV exposure (Table 1, Additional file 1). Successful tracing was defined as making contact with the MSP, regardless of whether they accepted HTS or not. This manuscript has been written according to the template for intervention description and replication (TIDieR) recommendations checklist (Additional file 2).

**Table 1:** Standard protocol for male sex partner tracing

Attempt	Mode	Timeframe for male sex partner tracing
1	Phone	Immediate (within seven days of female index enrolment)
2	Phone	Within seven days of the first attempt
3	Phone	Within seven days of the second attempt
4	Physical	Within seven days of the third attempt
5	Phone / physical	7–14 days after the fourth attempt
6	Phone / physical	7–14 days after the fifth attempt

### Study setting

This analysis was conducted within the aPS scale-up study, a collaboration between Kenya’s Ministry of Health’s National AIDS and STI Control Program (MOH NASCOP), PATH-Kenya, and the University of Washington (UW). It was conducted in 31 facilities in in two high-HIV prevalence counties (Kisumu and Homa Bay) in western Kenya in western Kenya [16] and had two aims: 1) to determine the effectiveness of aPS when integrated within routine HTS, and 2) to evaluate the implementation of aPS including the integration, implementation fidelity, acceptability, demand, and costs of the intervention [16, 17].

### Conceptual framework

**Fig. 2:**
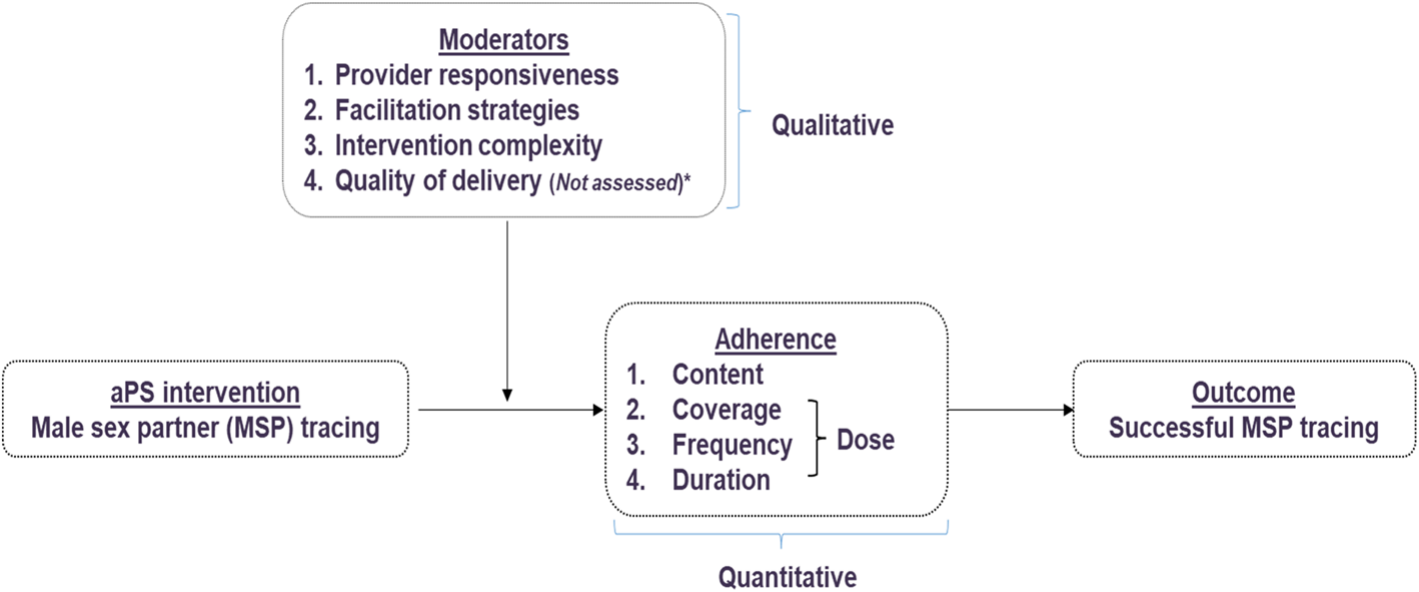
Modified conceptual framework for implementation fidelity. *Quality of delivery was not assessed using observation checklists due to COVID-19 social distancing restrictions

Using the implementation fidelity conceptual framework described by Carroll et. al (Fig. 2), we defined implementation fidelity as the extent to which HTS providers followed the protocol for phone and physical participant tracing [1]. We described components of its two key elements: adherence—the extent to which HTS providers offering aPS adhered to its protocol as outlined, and moderators—contextual factors influencing the extent of implementation fidelity to the intervention (Table 2).

**Table 2:** Operational definitions, types of data, and data sources for each construct in the modified conceptual framework

Construct	Operational definition	Type of data	Data source
**Adherence**			
Content	The core component of the aPS intervention was partner tracing defined as contact with male sex partners through phone call or physical tracing	Quantitative	Facility reports
Coverage	The number of individuals reached by aPS i.e., number of mentioned MSPs traced and notified	Quantitative	Facility reports
Frequency	The number of times providers attempted to contact partners by phone or physical tracing attempts	Quantitative	Facility reports
Duration	Time taken for the six tracing attempts as defined in the aPS protocol	Quantitative	Facility reports
**Moderators**			
Provider responsiveness	The extent to which HTS providers respond to, or are engaged by the aPS intervention	Qualitative	In-depth interviews
Facilitation strategies	Approaches to standardize and optimize fidelity to aPS, e.g., training, provision of manuals, monitoring, evaluation	Qualitative	In-depth interviews
Intervention complexity	Perceived clarity of the aPS protocol to the HTS providers	Qualitative	In-depth interviews
Quality of delivery	Degree to which aPS was delivered to achieve its intended purpose as assessed through direct observation	Not assessed due to COVID-19 social distancing restrictions	Not assessed

### Study procedures

In the aPS scale-up study, newly diagnosed HIV positive females (female index clients) were enrolled by HTS providers and their male sex partners (MSPs) were traced for HIV testing [16]. HTS providers were MOH-certified facility-based lay workers with diplomas in social science or counseling psychology and undergone three-week training on HTS. Providers offered HTS to clients at participating clinics, assessed eligibility, and offered aPS to eligible female index clients at the time of HIV diagnosis.

Consenting females were asked to provide names and contact information for all MSPs in the last 3 years. MSPs were then traced by HTS providers (provider referral) either on phone or physically, notified of their exposure, and tested for HIV. All HIV-positive MSPs were encouraged to enroll to the study while HIV-negative MSPs were counselled on HIV prevention strategies as per the national guidelines including consistent condom use and referral for pre-exposure prophylaxis. Enrolled participants were followed up at six weeks, six months, and 12 months post-enrollment to assess linkage to antiretroviral therapy (ART), intimate partner violence (IPV), and relationship dissolution, with viral load testing conducted at 12 months. Participants reporting history of IPV or relationship dissolution received counselling and were referred to the nearest gender-based violence counselor for further support.

### Study participants, sites

For the quantitative data, facility level phone and physical tracing records were collected by HTS providers from 31 facilities between November 2018 and December 2020. Tracing data were used to ascertain the coverage, frequency, and success of each tracing attempt overall and by county. Based on the study’s aPS tracing protocol (Table 1), at least 6 tracing attempts were to be made with the first three as phone attempts, and physical tracing attempted on the fourth try if the first three phone tracing attempts were unsuccessful.

For the qualitative data, we conducted 14 in-depth interviews (IDIs) with HTS providers each lasting between 60 to 90 min. The IDIs were conducted between May and August 2020. These providers were selected from 8 facilities that were sampled by criteria-based purposive sampling to maximize variation on patient volume—assessed by the number of female index clients tested for HIV, and aPS performance—assessed by MSP elicitation and enrollment [16, 18]. A semi-structured interview guide was developed using the conceptual framework on implementation fidelity to assess provider responsiveness, facilitation strategies, and intervention complexity [1].

### Data collection

Quantitative tracing data were collected using structured questionnaires administered on Android smartphones using open-source Open Data Kit platform [19]. Data on the phones were encrypted after collection and immediately transferred to a NASCOP server over a secured connection and backed up daily to a UW server [16].

Qualitative interviews were conducted by phone due to COVID-19 social distancing restrictions using either English, Swahili, or Luo. They were audio-recorded and transcribed from Swahili and Luo to English by an experienced qualitative interviewer (MO). Personal identifiers were removed from the recorded interviews and corresponding transcripts which were then assigned identification numbers.

### Data analysis

For the quantitative data, descriptive statistics were used to describe participant characteristics by county (Kisumu vs Homa Bay), type (phone vs physical), and success (successful vs unsuccessful) of tracing attempt. Categorical variables were described using absolute counts and proportions; continuous variables were described using medians and interquartile ranges (IQR). Pareto charts based were used to determine the tracing attempts at which most clients are successfully traced [20].

For the qualitative data, two independent coders, BMW and MO used thematic content analysis with both deductive and inductive coding to develop the codebook using key domains from the conceptual framework on implementation fidelity [1]. The codebook was tested and refined on two transcripts, then the remaining transcripts were coded using the finalized codebook that contained 33 codes. We then utilized memos to organize the codes into four themes and seven sub-themes. For any coding discrepancies, consensus was reached through discussion. Transcripts were analyzed using ATLAS.Ti version 8.4.4 and Microsoft Excel.

### Data integration

We applied a contiguous narrative approach where we reported the quantitative and qualitative findings as separate sections of the results [21]. Quantitative results were reviewed concurrently with the key emerging themes from the qualitative analysis and assessed for congruence or divergence using the modified conceptual framework for implementation fidelity. Both quantitative and qualitative data were given equal priority during analysis [15].

## Results

The study results were organized based on the elements in the conceptual framework for implementation fidelity (Table 2). The adherence section reflects the quantitative data, while the moderators section reflects the qualitative data.

### Adherence

#### Content of tracing attempt

**Fig. 3:**
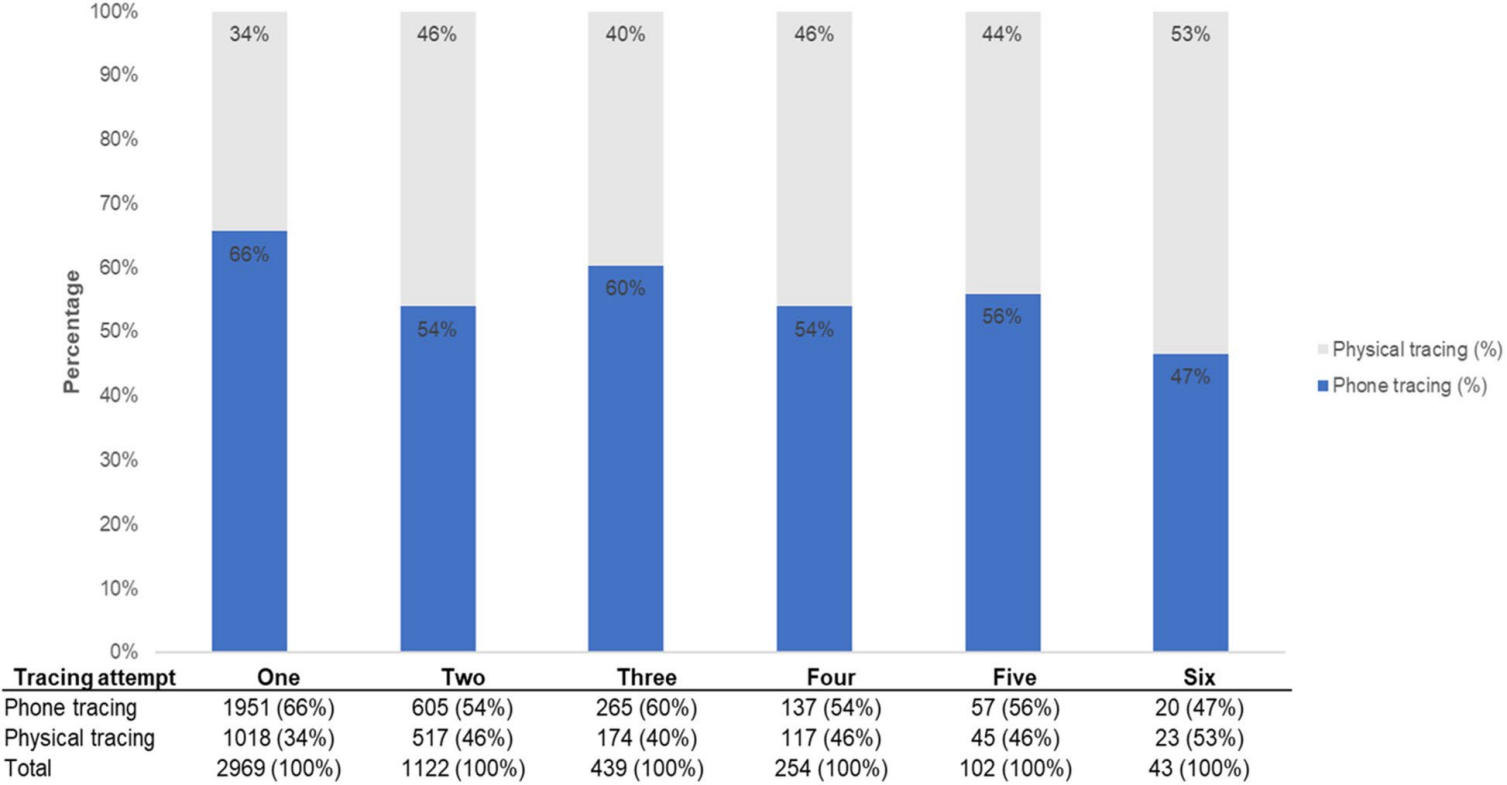
Overall proportion of phone versus physical tracing per attempt

The proportion of tracing attempts occurring by phone ranged from 47 to 66%, with the highest proportion occurring on the first attempt and lowest on the final attempt (Fig. 3). When compared to the tracing protocol (Table 1) where the first three tracing attempts were expected to be conducted only on phone HTS providers did not strictly adhere to the aPS protocol as prescribed (phone tracing: attempt 1 = 66%; attempt 2 = 54%; attempt 3 = 60%).

#### Coverage

Features of MSP tracing attempts, and characteristics of the HTS providers interviewed are presented in Tables 3 and 4, respectively. Overall, 3017 MSPs were mentioned by the female index clients, of whom 98% (2969/3017) had at least one tracing attempt (Table 3). Most partners were successfully traced (2831/2969, 95%). Although the study protocol recommended for three phone tracing attempts before physical tracing, phone tracing did not appear as successful in Kisumu and therefore HTS providers more frequently opted to physical tracing. More partners were successfully contacted in-person in Kisumu (Kisumu: 825/1496, 55% vs Homa Bay: 586/1473, 40%), while more partners were successfully contacted through phone calls in Homa Bay (Kisumu: 557/1496, 37% vs Homa Bay: 863/1473, 59%).

**Table 3:** Tracing of male sex partners overall and by county

	**Homa Bay**	**Kisumu**	**Overall**
Partner mentioned			
Male sex partner	1479 (100%)	1538 (100%)	3017 (100%)
Relationship type			
Sexual partner	1477 (100%)	1534 (100%)	3011 (100%)
PWID^a^ partner	2 (0%)	4 (0%)	6 (0%)
Traced	1473 (100%)	1496 (97%)	2969 (98%)
Successful: Phone tracing	863 (59%)	557 (37%)	1420 (48%)
Successful: Physical tracing	586 (40%)	825 (55%)	1411 (48%)
Not successful	24 (2%)	114 (8%)	138 (5%)
Not traced^b^	6 (0%)	42 (3%)	48 (2%)

^a^*PWID* Person who inject drugs

^b^Reasons not contacted—Physical locator information incorrect: 38% (18/48), mobile phone information incorrect: 25% (12/48), female index client brought MSP to the facility: 17% (8/48), MSP came to the clinic: 6% (3/48), other reasons not specified: 15% (7/48)

**Table 4:** Characteristics of HTS providers participating in the in-depth interviews

	**Homa Bay (*n* = 7)**	**Kisumu (*n* = 7)**	**Overall (*N* = 14)**
Age (years)			
Median (Range)	38 (31—52)	31 (25 – 38)	35 (25–52)
Gender			
Male	2 (29%)	2 (29%)	4 (29%)
Female	5 (71%)	5 (71%)	10 (71%)
Level of education			
Post-secondary education	7 (100%)	7 (100%)	14 (100%)
Location of facility			
Urban	3 (43%)	2 (29%)	5 (36%)
Rural	4 (57%)	5 (71%)	9 (64%)
Type of facility			
Public	7 (100%)	2 (29%)	9 (64%)
Faith-based	0 (0%)	5 (71%)	5 (36%)
Facility volume & performance			
High-volume high-performance	2 (29%)	4 (57%)	6 (43%)
High-volume low-performance	3 (43%)	1 (14%)	4 (29%)
Low-volume high-performance	1 (14%)	1 (14%)	2 (14%)
Low-volume low-performance	1 (14%)	1 (14%)	2 (14%)

Fourteen HTS providers, mostly female (10/14, 71%) with a median age of 35 years (range 25–52), participated in the IDIs (Table 4). All had post-secondary education, and most worked in high volume (10/14, 71%), rural (9/14, 64%), or public (9/14, 64%) health facilities.

#### Frequency of tracing

**Fig. 4:**
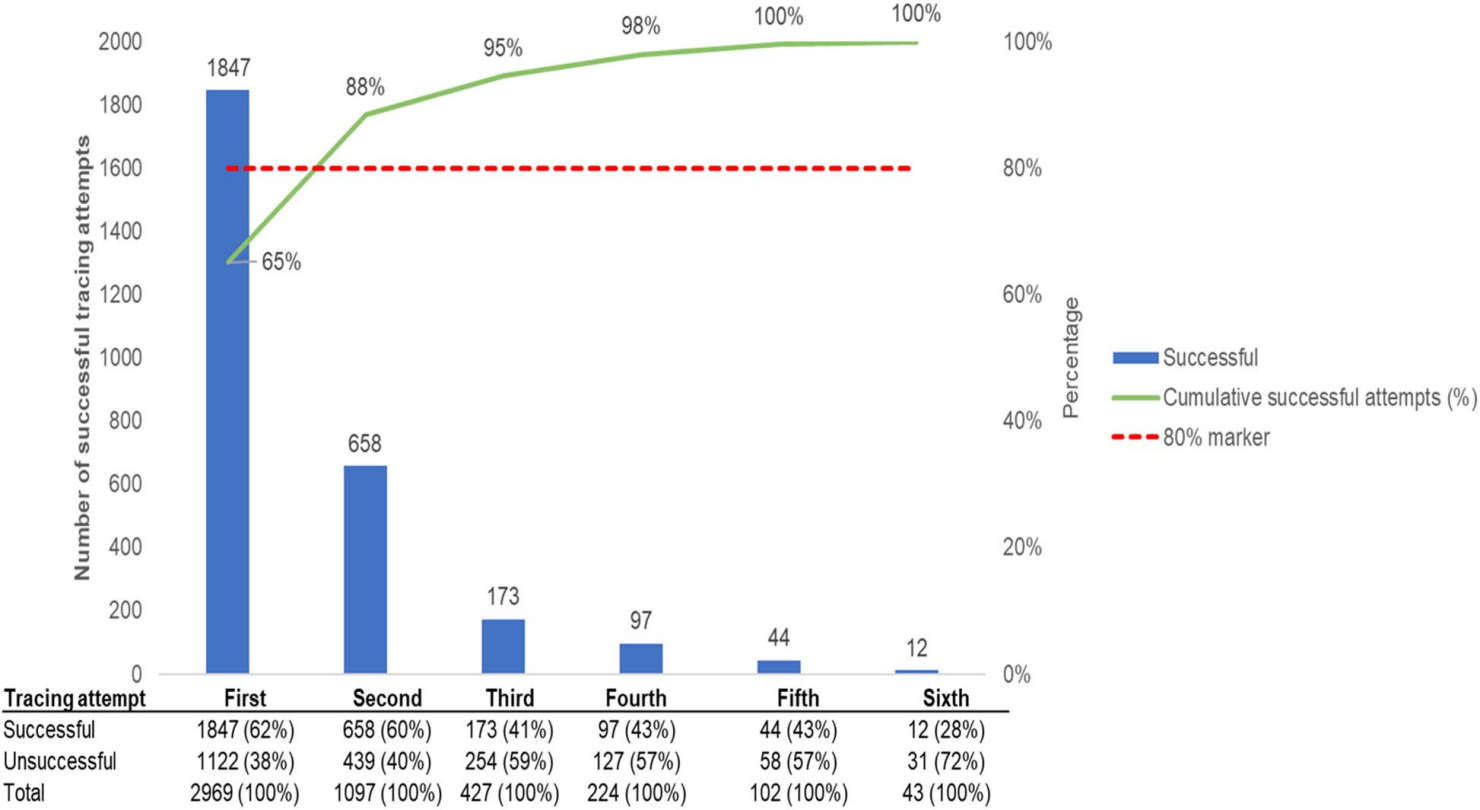
Pareto chart indicating overall success by tracing attempt. Missing data from 138 participants. Cumulative successful attempts (%) line uses 2831 as the denominator. The data table at the bottom of the figure uses the sum of successful and unsuccessful attempts at each of the six tracing attempts as the denominator

Two-thirds of MSPs were successfully traced at the first attempt, while the first two tracing attempts accounted for almost 90% of all successful tracing attempts (Fig. 4). Success of tracing attempts gradually declined from the first (62%) to the sixth (28%) tracing attempt.

#### Duration

The timing for each of the six attempts is as per study protocol.

### Moderators

**Fig. 5:**
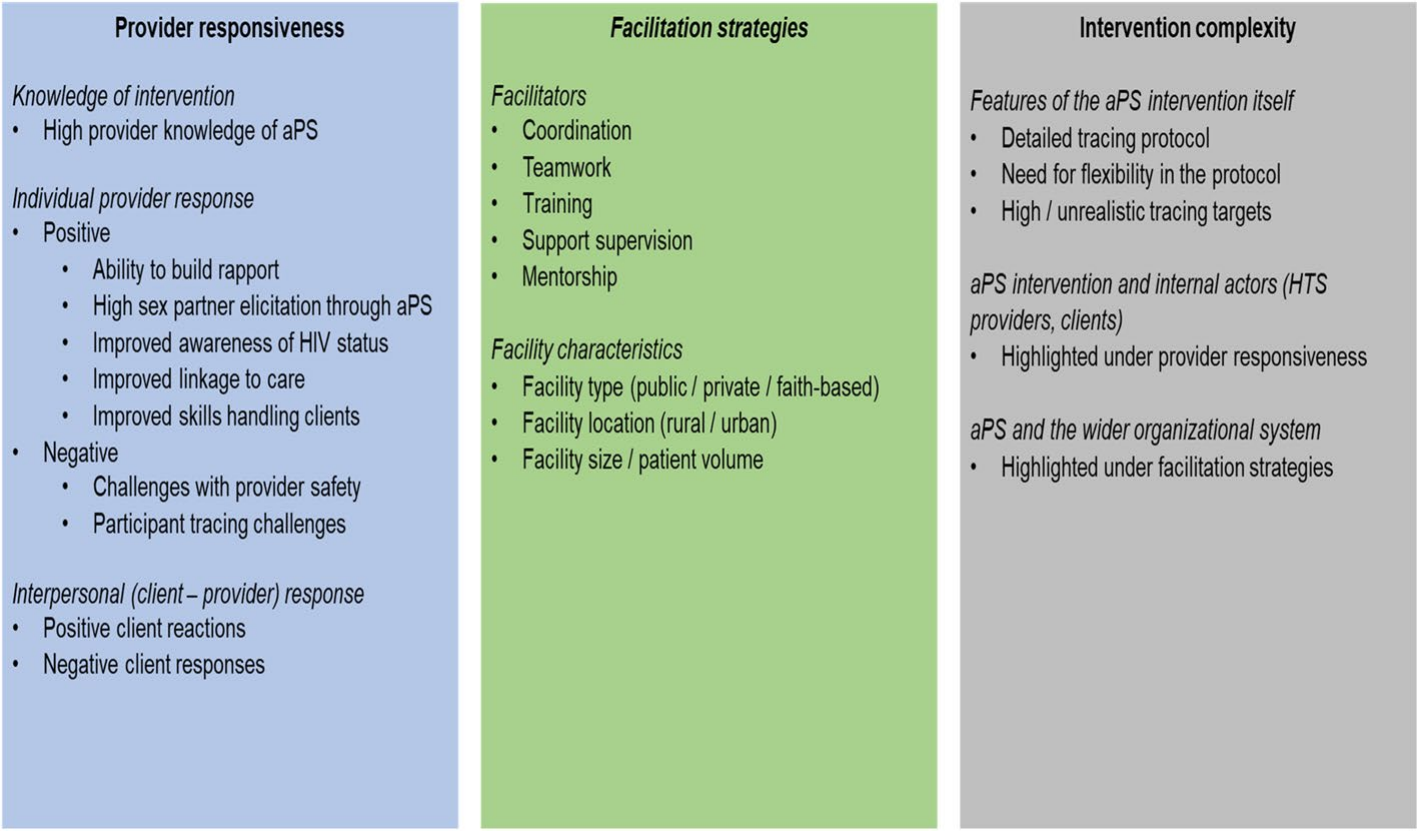
Moderators influencing implementation fidelity to aPS. Positive and negative provider responses to aPS had an impact on perceived complexity due to interactions between internal actors (clients and providers). Facilitation strategies, contributed by the wider HTS organizational system, also affected the perceived complexity of aPS

The main themes for provider responsiveness, facilitation strategies, and intervention complexity are summarized in Fig. 5. Individual (provider), interpersonal (client—provider), and health systems (facility, organization) level interactions appeared to affect implementation fidelity to aPS.

#### Provider responsiveness

Provider response to aPS was largely positive. The themes, subthemes and exemplar quotes are presented in Table 5. At an individual level, most providers were very knowledgeable of aPS, articulated its processes and protocol well, and had good skills to build rapport and trust with MSPs which positively impacted implementation fidelity. However, challenges during implementation impeded fidelity to the protocol. These include physical tracing challenges such as navigating difficult terrain, poor weather, and inadequate locator information (e.g., incorrect / incomplete participant names, incorrect / non-functioning phone numbers, inaccurate home addresses), and safety concerns including verbal abuse during phone tracing, threats of physical harm, and suspicion of being sexual partners to participants.

**Table 5:** Themes on provider responsiveness

Themes	Sub-themes	Exemplar quotes
**Knowledge of aPS**	Knowledgeable	" Sometimes you call and the client tells you I am busy, that one you still document. But it should be at least three or four times before you make a physical attempt." KII 017
**Individual provider response to aPS**	Positive: Building rapport	" At the start of counseling, testing and linkage to care, you have created good rapport with this client, (and) you will be a king or a queen to (them) concerning their health especially on HIV care because she or he will say that the first person who contacted him inside the health facility for her treatment was you." KII 019
	Positive: High sex partner elicitation	" I like aPS because it gives us so many SPs (sexual partners).… You know after index gives you like 4, 5 sex partners, it will help you get more. So, you know if you go and test like all those 5, about 3 or 2 must be HIV positive and that helps us make them know their status so that you can make them start taking medicine." KII 020
	Positive: Improved awareness of HIV status	"Another thing is that you are going to help this client because if the clients know their HIV status early enough. Sometimes, the client had not planned to go to the facility for testing but now you have gone to them and they are tested early enough, you see they will start treatment early enough in case they are positive and in case they are negative then we shall teach them how they can take care of themselves." KII 027
	Positive: Improved linkage to care	"So, the moment you identify somebody is positive, putting this person on care early you prevent a lot of issues like opportunistic infections or other complications that might come as a result of not seeking treatment in time.” KII 016
	Positive: Improved provider skills	"Interviewer: What do you like about doing provider referral in particular? Provider: (light laugh) It is good because it makes you gain some knowledge and tricks of getting to the person, you must use your tricks on how to meet the person. (You ask yourself) “How will I meet this person? What will I do? In such and such a situation, what do I do exactly so that I may reach this person?” KII 028
	Negative: Challenges with provider safety	"Another bad experience, I was chased by a machete. If it was not for my motorbike, I don’t know how that could have gone. I had my motorbike with me. That has always been my rule. I leave my motorbike on at the front of the house with a very low sound. I finish with you and take off immediately. It is always left on when I go for tracing and don’t turn off the key." KII 022 "I am saying that the client can tell you to go to his/her place to do the test and maybe when you get there the client has a different agenda towards you. Maybe he wanted to seduce you or maybe he wants to touch you suggestively or maybe he wants to rape you. Things like that, those are some of the challenges that are there" KII 030
	Negative: Tracing challenges	"You find that it is also hard in that during the physical tracing, you need to go out and the sun is so scorching and you have to walk very far where the motorbikes cannot reach" KII 021
**Interpersonal (client – provider) response to aPS**	Positive: Client’s positive reactions	"Last month I got a female positive enrolled for the first time. She had issues with the drugs she was given. Instead of talking to the clinician who served her she came to consult with me…On her next appointment when she came back to the clinic, she came straight to me without going to the CCC where she had the appointment….She said she was scared to talk to the clinician who gave her the drugs. What I discovered with that story is that the first encounter with a client is the one that will decide whether they will open up and trust you or not." KII 019
	Negative: Client’s negative responses	"The experience has been good but sometimes you find some clients who are harsh. They will start asking you, where did you find my number, who are you and you know some are harsh. But for you, just relax and talk slowly and maybe…. you don’t lose hope. You keep calling and maybe after two three days the client will agree." KII 020

At an interpersonal level between MSPs and providers, positive MSP responses to early notification, HIV testing, and notification made providers respond favorably to aPS, thus motivating them to adhere to the protocol. On the other hand, negative MSP responses made it difficult for HTS providers to maintain fidelity to the aPS protocol. For instance, some MSPs required additional time before being ready to receive aPS due to fear of disclosure and concerns over privacy and confidentiality. Other MSPs preferred HTS providers of a particular gender and age. For instance, some older male MSPs preferred older / male HTS providers and would, therefore, be less forthcoming with young / female HTS providers.

#### Facilitation strategies

Key facilitation strategies noted included coordination, teamwork, training, and support supervision (Table 6). HTS providers were keen to work in environments that embodied these characteristics and welcomed open learning and sharing which helped them maintain fidelity to aPS. Facility characteristics were also noted to have an impact on implementation fidelity. Some HTS providers preferred working in public facilities due to larger volumes of clients. Others preferred rural facilities where clients were easier to trace as they did not change domicile as frequently as those in urban areas. There were differing opinions on the impact of facility characteristics on implementation fidelity where some providers felt that they had no impact, while others felt that it had an indirect impact. For instance, providers felt that implementation fidelity was easier in larger volume public facilities where more healthcare services were available. MSPs were more likely to agree to come to these facilities for HTS as they could then seamlessly continue with care at the same facility.

**Table 6:** Themes on facilitation strategies

**Theme**	**Sub-theme**	Exemplar quotes
**Facilitators**	Coordination & teamwork	"The difficult ones one, like I said earlier, I will just refer to the next counselor so that he/she can also try because remember maybe the skills that I am using is not the same skills that the other person is going to use and that has really helped us because where I work, we have varied age brackets." KII 023
	Training	"And then we also have the regular refresher courses. Some of these we do them internally, we share experiences why is somebody succeeding and you are not succeeding. We compare the notes and then we come up with ways of consolidating our working experiences." KII 016
	Support supervision	“Yes, support supervision also helps a lot. You know when we go there you open up and share the challenges you are facing with the clients, the experiences of the other HTS providers, how better others are doing it so that you can also do and achieve. Yes. When you get a colleague being praised for doing a commendable job and you are doing the same thing, you can share in these forums and get to know whatever this person is doing differently that you can also do." KII 028
**Facility features**	Facility type (public / private / faith-based)	"Interviewer: …What about the government versus private hospital or mission hospital? Which one is easier? Provider: I think government is better being that many people know that some services are offered for free but in private hospital you will be charged most of the services." KII 027
	Facility location (rural / urban)	"Interviewer: Okay, what about the facilities in urban versus rural areas, which one is easier to work in for aPS? Provider: Rural Interviewer: Why? Provider: Because in rural, when a client tells where the home is then you will get him/her but in urban mostly clients stay in rentals. Sometimes somebody can tell you “Come next week” when you go you find that the person has moved. But with rural if you’re given locator (information) and you are told where the home is then you are sure you will get the client or you will find the person who knows the client" KII 030
	Facility size / patient volume	"I think in bigger facilities like the sub county hospitals it (*aPS*) can work better. You see most people like bigger facilities so if you tell them that you are calling from [county hospital name], compared to someone who calls and says the call is from a dispensary then you will find that they will prefer to come to [county hospital name] because that is where they will get many services. " KII 027 "Ok, the size of the facility may not affect the performance, provided that the facility has organized itself well; the staff working in the facility. If there is teamwork in the facility, even if the facility is small, then work will be seen." KII 026

#### Intervention complexity

Factors that contributed to intervention complexity of aPS included: 1) features of the aPS intervention, 2) unpredictable client-provider interactions, and 3) interactions between the intervention and the healthcare system (Table 7). As to the features of the aPS intervention, HTS providers knew the correct aPS protocol, were well versed with its components, and were aware of the frequency and intervals between tracing attempts. However, some felt that flexibility in the protocol was required especially when tracing difficult participants who would require additional tracing attempts. Secondly, unpredictable client-provider interactions, whose main themes are mentioned earlier under participant responsiveness, led to intervention complexity. Unlike client-initiated HIV testing, HTS providers offering aPS often found themselves working with clients or their families who were not ready or willing to be contacted for HTS. Thirdly, intervention complexity was introduced during provider interactions with the healthcare system whose main themes were outlined under facilitation strategies. For instance, challenging work environments were seen to negatively affect fidelity to the protocol.

**Table 7:** Themes on intervention complexity

Theme	Sub-theme	Exemplar quote
**Features of the aPS intervention**	Tracing protocol	"If I get a client today, let me say now, I talk to them then agree. We agree with the client on when to start it. A client may give you two to three weeks which might be so long so according to me this is what I do, after I have tested client today, I may start it immediately or after two or three days. I’ll first make a call to this client then if I don’t reach the client, I will still do the phone calls- Three attempts. When it fails, now I start the physical tracing, I myself." KII 026
	Flexibility in conducting aPS	"Interviewer: So, is that *(aPS protocol)* sufficient or you think that needs to be improved? Provider: That one needs to be improved because aPS is supposed to be a process and clients are unique. You can’t deal with one client the same way you deal with maybe the other client; they are unique. " KII 030
**aPS and internal actors (HTS providers, clients)**	Individual provider, and client-provider interactions	Quotes highlighted under provider responsiveness
**aPS and organizational system**	aPS and the healthcare system	Quotes highlighted under facilitation strategies

## Discussion

This is one of the few studies in Africa assessing implementation fidelity to HTS related interventions. We assessed adherence to the aPS protocol by the content—tracing attempts, coverage—number of MSPs traced of those mentioned, frequency – number of times each MSP was traced, all based on the duration of the intervention – prespecified by the timepoints in the protocol. We observed highly successful partner tracing where almost all mentioned MSPs were traced. However, HTS providers did not strictly adhere to the prescribed timing of the phone and physical tracing attempts. Though we expected that the first three tracing attempts would be on phone, we found that almost half of them were conducted in-person, potentially due to HTS providers not recording unsuccessful tracing attempts or due to inadequate MSP contact information.

The first two tracing attempts were critical with almost 90% of MSPs successfully traced in these two attempts. However, the success of each subsequent tracing attempt declined over time from about 60% at the first attempt to less than 30% by sixth tracing attempt. This signifies the importance of creating immediate rapport with MSPs during the initial phone and physical tracing attempts. There’s also need to retain open lines of communication with female index clients who can provide get additional tips on how best to contact elicited MSPs. In a cluster randomized control trial on aPS in Kenya, HTS providers with great interpersonal skills and capacity to create trust and strong bonds with the participants were able to get additional information on the sex partners that was crucial for tracing and successful notification [22]. This indicates the importance of continuous training and mentorship of HTS providers on communication and interpersonal skills with clients.

We assessed moderators to implementation fidelity namely provider responsiveness, facilitation strategies, and intervention complexity. These contextual factors impacted fidelity to the aPS protocol at the individual (provider), interpersonal (client-provider), and health system (facility, organizational) levels. Most HTS providers responded positively to the aPS intervention as they could more easily create rapport with clients. However, some found it challenging to adhere to the protocol due to safety concerns for both clients and providers, as well as challenging tracing environments, similar to findings from other aPS studies [22–24, 12, 25–27]. In a systematic review of aPS programs, authors recommended that the potential harm arising from disclosure through aPS needed to be balanced against the benefit of diagnosing HIV infection and linking people to treatment [12]. Similarly, in a review of aPS implementation in Kenya, Cameroon and Mozambique, stakeholders recommended continual revision of aPS curricula based on the context and ongoing monitoring and evaluation to ensure safety and sustainability of the intervention [24]. Program implementers will need to constantly reassess the aPS program and provide necessary support to both staff and clients to ensure intended outcomes are achieved with minimal risk of harm. Strategies to improve provider safety mentioned during the interviews included pairing providers with clients of similar age and gender; encouraging providers to meet with clients in public settings whenever possible; and pairing providers with other providers or CHVs during participant tracing.

Facilitation strategies at the organizational level such as coordination, teamwork, training, and support supervision promoted implementation fidelity to aPS. In a Ghana study evaluating the integration of national TB screening guidelines in HIV clinics, ongoing training of health providers on implementing all components of the guidelines was recommended as a means to improve implementation fidelity [4]. In a project reviewing social and behavior change communication to improve childhood vaccination in India showed that the commitment of the implementers and periodic meetings with supervisors contributed to high levels of implementation fidelity [28]. Such strategies at the healthcare system level will go a long way in improving implementation fidelity to aPS.

HTS providers did not strictly adhere to the aPS protocol given the intervention’s complexity with some suggesting increased flexibility especially when dealing with difficult individuals. We observed that in Kisumu, a largely cosmopolitan county, MSPs were harder to trace on phone as they frequently changed their locator information similar to other findings [18]. Hence, they were more likely to be traced in-person at the first tracing attempt. This highlights the need to evaluate and adapt aPS approaches based on the norms of communities served. In a qualitative analysis of fidelity to an evidence-based HIV prevention in the US, providers viewed program manuals as guides rather than static texts with some viewing the prescriptive nature of manuals as undermining their efforts to fully engage with participants [29]. In a review of implementation fidelity to HIV self-testing, the overly complex instructional materials were seen as significant impediments to adaptation [30]. Adaptations to intervention protocols will require input from key stakeholders to ensure that the protocols accurately reflect the community context and adequately cater to client needs.

Our study had several strengths. First, we applied a modified implementation fidelity framework validated for use in complex health programs. This enabled us to holistically evaluate contextual factors affecting implementation fidelity to aPS and identify areas for improvement. Second, we collected and analyzed both qualitative and quantitative data which enabled us to triangulate our results and give a deeper meaning to our findings. Third, our analysis was conducted in pragmatic settings making our results generalizable to programs within similar settings within and outside Kenya.

We noted several limitations in our study. First, the focus of our quantitative results was limited to tracing attempts. We did not evaluate HIV counselling, testing, referral, or linkage to care which may have impacted implementation fidelity. Second, we were not able to directly observe quality of delivery or conduct face-to-face interviews due to COVID-19 social distancing restrictions that restricted in-person interactions. While this may have led to loss of visual cues, it may have allowed respondents to more freely disclose sensitive information that they may not have otherwise done in-person [31]. Third, we only offered provider referral in our study which might have made it more challenging to trace participants requiring a more flexible tracing approach. Finally, we relied on self-reported tracing attempts from the HTS providers and did not record the actual timing for each attempt which may have led to recall and social desirability bias. Future research should consider technologies that support real time tracking of phone calls and in-person interactions with participants.

## Conclusion

In summary, our study systematically assessed implementation fidelity to aPS in western Kenya. Despite highly successful partner tracing, contextual factors at the individual (provider), interpersonal (client—provider), and healthcare system (facility) levels either facilitated or impeded implementation fidelity to the aPS protocol. As MOHs around the globe prioritize strategies to reduce new HIV infections, our findings highlight the importance of conducting fidelity assessments during the scale-up of interventions.

## Supplementary Information

**Additional file 1. **Partner tracing standard operating procedures.

**Additional file 2. **Template for intervention description and replication (TIDieR) checklist.

## Data Availability

The datasets used and/or analyzed during the current study are available from the corresponding author on reasonable request.

## Abbreviations

aPS: Assisted partner services
ART: Antiretroviral therapy
HTS: HIV testing service
IDI: In-depth interview
IPV: Intimate partner violence
MOH: Ministry of Health
MSP: Male sex partner
NASCOP: National AIDS and STI Control Program
PLWH: People living with HIV
PWID: Persons who inject drugs
TIDieR: Template for intervention description and replication
UW: University of Washington
WHO: World Health Organization
