# Supplementary material for: Implementation fidelity to HIV assisted partner services (aPS) during scale-up in western Kenya: a convergent mixed methods study
Source: BMC Health Serv Res. 2023 May 19;23:511. doi: 10.1186/s12913-023-09541-1 (PMC10198752; doi:10.1186/s12913-023-09541-1)
Supplement: Supplementary file 1 — Additional file 1. Partner tracing standard operating procedures. [file 12913_2023_9541_MOESM1_ESM.docx]

**PARTNER TRACING STANDARD OPERATING PROCEDURE**

**Purpose**

To guide the HTS counselors on how to document and conduct partner tracing for assisted partner notification services (aPS).

**Scope**

This standard operating procedure (SOP) is relevant to all field staff who are involved in client tracing for aPS.

**Materials Required**

- Partner Contact Form (paper-form)
- Client Tracing Form (electronic)
- Oral Script for Phone Contact Tracing (paper-form)
- Oral Script for In-person Tracing (paper-form)
- Partner Tracing Summary Form (electronic)

**Procedures**

At enrollment, index clients will elicit their sexual partners. HTS counselors will then contact these partners and notify them of HIV exposure by phone or through an in-person visit.

In summary, partners will first be contacted by phone up to 3 times. If the partner cannot be located by phone, the HTS counselor will trace the partner physically once using the locator information written in the “PARTNER CONTACT FORM.” If the four initial attempts i.e., three phone contacts and one physical tracing are unsuccessful, two additional tracing attempts will be made. The mode of tracing (phone vs. in-person) of these last two attempts will be determined by the HTS counselors on a case-by-case basis.

All attempts to contact sexual partners must be documented on the client tracking logbook and the client tracing form on paper with date, time, and description of tracing attempt. **Electronic client tracing form in ODK must also be completed for all partner tracing attempts**.

Phone tracing:

1. Partner’s telephone numbers can be found on the “PARTNER CONTACT FORM,” which is located in the index’s study folder.
   1. NOTE: “PARTNER CONTACT FORM” contains partner’s telephone numbers and locator information.
2. HTS counselors will make the first attempt to contact the partner by phone **on the day** of index client enrollment. The first attempt must be made no later than 1 week from index client enrollment.
3. Before calling the partners, HTS counselors will have the study tablet, oral script for phone contact tracing (see Appendix), and “PARTNER CONTACT FORM” ready.
4. Using the telephone numbers located in the “PARTNER CONTACT FORM,” HTS counselor will make the phone call.
5. IF THE PHONE IS ANSWERED:
   1. If the phone is answered, use the oral script to confirm if the right person is on the line.
   2. After confirming that it is the partner who is on the line, the HTS counselor will follow the oral script to 1) introduce himself/herself as a Ministry of Health worker, 2) notify the partner of HIV exposure, and 3) schedule an HIV test.
      1. In phone tracing, it is important to deliver the following key messages to the partners:
         1. HIV is a sexually transmitted disease
         2. Partner’s exposure to HIV
         3. Importance of HIV testing and knowing his/her own HIV status
         4. Prevention measures
         5. Scheduling an HIV test
      2. On the “CLIENT TRACING FORM” in the study tablet:
         1. Check “Yes” to the “Was the client located” question
         2. Check “By telephone” to the “How was the client contacted” question
         3. Answer the rest of the questions on the form accordingly
   3. If the partner inquires how their contact information was acquired, HTS counsellor will inform them that they are not allowed to disclose that information
      1. If the partner insists or threatens the HTS counselor, they will inform them that it was provided by a person that cannot be revealed since they are bound with confidentiality
   4. If the partner agrees to come for testing, the HTS counselor will let him know their (HTS counselor) name, health facility, and when they can be found at the facility
   5. If the partner answers the phone but indicates that it is not a good time to discuss his/her health matter, the HTS counselor will re-schedule a phone call at a time the partner suggests. The HTS counselor will record the time that s/he will make the next attempt to reach the partner in the “comment” section.
      1. On the “CLIENT TRACING FORM” in the study tablet:
         1. Check “Yes” to the “Was the client located” question
         2. Check “By telephone” to the “How was the client contacted” question
         3. Check “Yes” to the “Did you notify the client of their exposure to HIV?” if the message was delivered; check “No” otherwise
            1. If “No,” write down why the partner was not notified of HIV exposure
6. IF THE PHONE IS NOT ANSWERED:
   1. If the phone is not answered, the HTS counselor/HA will record the time that s/he will make the next attempt to reach the partner in the “comment” section.
      1. On the “CLIENT TRACING FORM” in the study tablet:
         1. Check “No” to the “Was the client located” question, and write down why the client was not located
         2. Check “By telephone” to the “How was the client contacted” question
7. The HTS counselor will make up to 3 phone tracing attempts; all attempts must be documented accurately in the “CLIENT TRACING FORM.”
8. It is important to monitor partners’ scheduled dates for HIV testing. If partners do not contact HTS counselor or come in for HIV testing on the scheduled dates, HTS counselors must make a follow-up call immediately to re-schedule.
9. If all three phone tracing attempts are unsuccessful, an offsite physical tracing will be conducted within **a week of the 3^rd^ attempt**.
10. The in-person tracing script (Appendix 1) will guide the HTS counselor to do physical tracing.

In-person tracing:

1. Offsite physical tracing occurs when 3 phone tracing attempts fail. It must happen **within a week** from the last phone tracing attempt. Using the locator information provided by the index client, the HTS counselor will trace the partner in-person. Partner’s locator information can be found in the “PARTNER CONTACT FORM.”
2. Before conducting physical tracing, the HTS counselor will notify the Site Study Coordinator of the physical tracing plans and obtain money for transportation.
3. The “PARTNER CONTACT FORM” must remain at the facility and cannot be taken out to the field when tracing a partner. HTS counselors may use their notebooks to record minimal information that is needed for partner tracing. Any confidential information, particularly their medical history, cannot be written on the notebook.
   1. NOTE: if the notebook is lost, the HTS counselor must notify the Site Study Coordinator immediately.
4. For physical tracing, the HTS counselor will carry the study tablet, a notebook, oral script for physical tracing, and a copy of index barcode. S/he must inform a fellow HTS counselor where s/he plans to go for partner tracing. This is important to ensure staff safety.
5. IF THE PARTNER IS LOCATED:
   1. Once you locate a partner physically, use the oral script (see Appendix 2) to 1) introduce himself/herself as a Ministry of Health worker, 2) obtain written consent for study participation, 3) notify the partner of HIV exposure, and 4) schedule an HIV test.
      1. Written consent for study participation must be obtained from all partners, including known HIV-positives (KP’s), before HIV testing. However, a refusal to participate in the study will not affect their right to test for HIV or receive aPS if test positive.
      2. If the partner is a known HIV-positive, the HTS counselor will explain the importance of notifying his/her sexual partners of exposure to HIV and linking into care. KPs are also eligible for study participation.
         1. On the “CLIENT TRACING FORM” in the study tablet:
            1. Check “Yes” to the “Was the client located” question
            2. Check “In-person” to the “How was the client contacted” question
            3. Answer the rest of the questions on the form accordingly
   2. If the partner is located but states it is not a good time to discuss his/her health matter, the HTS counselor will re-schedule another phone call or in-person visit at a time the partner suggests. The HTS counselor will record the time that s/he will make the next attempt to reach the partner in the “comment” section.
      1. On the “CLIENT TRACING FORM” in the study tablet:
         1. Check “Yes” to the “Was the client located” question
         2. Check “In-person” to the “How was the client contacted” question
         3. Check “Yes” to the “Did you notify the client of their exposure to HIV?” if the message was delivered; check “No” otherwise
            1. If “No,” write down why the partner was not notified of HIV exposure
6. IF THE PARTNER IS NOT LOCATED:
   1. If the partner is not located with the physical tracing, the HTS counselor will record the time that s/he will make the next attempt to reach the partner in the “comment” section.
      1. On the “CLIENT TRACING FORM” in the study tablet:
         1. Check “No” to the “Was the client located” question, and write down why the client was not located
   2. Check “In-person” to the “How was the client contacted” question
   3. Contact index client to confirm whether the locator information provided is correct. If different, please make a note to the “PARTNER CONTACT FORM” (paper-based) and add it to the “comment” section in ODK client tracing form.

Two additional tracing attempts:

1. If all three phone tracing attempts and one physical tracing attempt fail, two additional tracing attempts will be conducted. Whether these two attempts will be done by phone or through an in-person visit will be determined by the HTS counselor on a case-by-case basis.
2. Partners will be considered lost-to-follow-up if they fail to test and enroll into the study after the 6 attempts that are within 3 months since index client enrollment; in this case, the “PARTNER TRACING SUMMARY FORM” will be completed.

**Special circumstances during partner tracing**

1. PARTNER DOES NOT HAVE A VALID PHONE NUMBER:
   1. If the index client did not provide a valid telephone number to reach the partner, the HTS counselor will contact the index client again to obtain the correct telephone number. This must be done **on the same day** that the telephone number is found invalid or missing.
      1. If an index cannot be reached or is unreachable due to either relocation or changes in phone numbers, contact the “ALTERNATIVE CONTACT PERSON” provided in the “INDEX CONTACT FORM” to obtain the correct phone number.
      2. If no alternative contact person is provided and index did not provide consent for HTS counselor to call the workplace, physical tracing should be planned
   2. The HTS counselor will ask the index client to provide correct telephone number as soon as possible, preferably within a few days.
   3. The HTS counselor follow up with the index client for up to 4 weeks to obtain the correct telephone number.
   4. If the index client says she does not have access to the partner’s correct telephone number, the HTS counselor will move onto physical tracing immediately. The first physical tracing attempt must be made **within 1 week** of the verification.
   5. If the first physical tracing is unsuccessful, two additional physical tracing attempts will be scheduled in one week apart.
   6. If all three physical tracing attempts are unsuccessful, the partner will be categorized as lost-to-follow-up (LTFU) and “PARTNER TRACING SUMMARY FORM” will be completed to close the case.
2. PARTNER HAS MISSING OR INVALID PHYSICAL LOCATOR INFORMATION:
   1. After the initial three unsuccessful phone tracing attempts, the partner must be traced physically. However, if the index client did not provide a valid locator information to reach the partner, the HTS counselor will contact the index client again to obtain the correct locator information. This must be done **on the same day** that the locator information is found invalid or missing.
   2. The HTS counselor will ask the index client to provide correct locator information as soon as possible, preferably within a few days.
   3. The HTS counselor will follow up with the index client for up to 4 weeks to obtain the correct locator information. Meanwhile, two additional phone tracing attempts will be made to reach the partner.
   4. If the index client says she does not have access to the partner’s locator information and the partner has already been contacted 5 times by phone and in-person unsuccessfully, the partner will be categorized as “lost-to-follow-up” and “PARTNER TRACING SUMMARY FORM” will be completed to close the case.
   5. If the correct locator information is obtained from the index client, the partner will immediately be traced in-person.
   6. If the first physical tracing is unsuccessful, two additional physical tracing attempts will be scheduled in one week apart.
   7. If all three physical tracing attempts are unsuccessful, the partner will be categorized as “lost-to-follow-up” and “PARTNER TRACING SUMMARY FORM” will be completed to close the case.

**APPENDIX 1: INTRODUCTION SCRIPT TO PARTNERS**

**1. PHONE CONTACT TRACING**

Hello, am I speaking with Ms/Mr.____________?

**[IF NOT]** Is Ms/Mr.____________ available?

**[If Partner is not available]** Thank you. I will try again later.

**[If YES]** I am ____________, a counselor/healthcare provider in ___[County]___. I have some important information for you. Is this a good time to talk? This will not take long and I assure you that our discussion will be confidential.

**[IF NO]** When would be a better time for me to call you?

**[If YES]** I am calling from the partner notification services Ministry of Health Program. It is a public health service to provide HIV testing and counseling to people who are exposed to HIV, which is primarily sexually transmitted. I am calling you today because we have recently learned that you might have been exposed to HIV. This does not mean you are infected; it just means that you have been exposed to HIV and will need to receive a HIV test to find out what your status is.  We cannot disclose how we learned about the exposure due to the need to maintain confidentiality. But, we would like to help you test for HIV for your own health. *(If partner inquires how their contact information was acquired or if the partner insists or threatens you, inform them that it was provided by a person that can not be revealed since you are bound to confidentility)*

If you are HIV-negative, we can give you information on how you can remain free from HIV. If you are HIV-positive, we can give you medicines to treat your HIV. These medicines will help you live a long life and reduce your chance of passing HIV onto others.

HIV testing services are available at health facilities Monday - Friday from 8:30 in the morning until 5:00 in the evening. Alternatively, we can send a counselor out to your home for an HIV test. Which option would you prefer?

**[Facility Test]** Which facility would you like to go for an HIV test? What day would you like to come?

**[Off-facility Test]** What date and time would you prefer for the counselor to come to your home for an HIV test?

Thank you.

**2. IN-PERSON CONTACT TRACING**

Hello, I am looking for Ms/Mr.____________. Is he/she around?

**[IF NOT]** Okay, thank you. Do you know when he/she will be back?

**[Once Partner is in front of you]** My name is____________, and I am a counselor/healthcare provider in ___[County]___.  Is there a private place that we can talk?

**[If YES]** I am from the partner notification services Ministry of Health program. It is a public health service to provide HIV testing and counselling to people who are exposed to HIV which is primarily sexually transmitted. I am here today because we have recently learned that you might have been exposed to HIV.  This does not mean you are infected.  It just means that you have been exposed to HIV and will need to receive a HIV test to find out what your status is.  We cannot disclose how we learned about the exposure due to the need to maintain confidentiality. We would like to help you test for HIV for your own health.

If you are HIV-negative, we can give you information on how you can remain free from HIV. If you are HIV-positive, we can give you medicines to treat your HIV. These medicines will help you live a long life and reduce your chance of passing HIV onto others.

I can test you for HIV right now. Would you like to do the test today?

**[If YES] ****Provide pre-test counseling, obtain informed consent, and do post-test counseling according to national HTS guidelines.

**[If NO]** If you prefer, you can come to __[Facility Name]_____ for an HIV test. HIV testing services are available Monday - Friday from 8:30 in the morning until 5:00 in the evening.

Would you like to go today? If so, I can accompany you to the clinic. If not, what day would you like to come to the health facility for an HIV test? I advise that you do the test at your earliest convenience. Please look for me when you get to ___[Facility Name]_____ for an HIV test.

If you test positive on your HIV test, I will also provide assisted partner notification services.   As the first part of the service, we will ask you to share with us the contact information of your sexual partners from the past three years. Then, either HIV testing services (HTS) Counselors or Health Advisors will attempt to contact these partners by phone or off-site visit to tell them they may have been exposed to HIV. We will keep your identity confidential while telling your sexual partners of their possible exposure. This means they will not tell your sexual partners how or who may have exposed them to HIV. However, in some cases it may be possible for them to figure out who referred them. If your partner(s) decide they want to take an HIV test and test positive, we will help them linked to care. Assisted Partner Services are important so that your partners can know their HIV status and take preventive measures accordingly to live a healthy life.

Do you have any questions? Would you like to receive aPS if you test positive?

**[Off-Facility Test] ****Provide pre-test counseling, obtain informed consent, and do post-test counseling according to national HTS guidelines

Thank you.

**APPENDIX 2: PARTNER TRACING SOP SUMMARY TABLE**

**Standard timeline for contact tracing**

| **Tracing attempt** | **Mode of tracing** | **Timeframe for tracing** |
| --- | --- | --- |
| Attempt 1 | Phone | Immediately; must be completed within 7 days after index enrolment |
| Attempt 2 | Phone | 24 hours up to 7 days after Attempt 1 |
| Attempt 3 | Phone | Up to 7 days after Attempt 2 |
| Attempt 4 | Physical | Up to 7 days after Attempt 3 |
| Attempt 5 | Phone/physical | 7-14 days after Attempt 4 |
| Attempt 6 | Phone/physical | 7-14 days after Attempt 5 |
| *NOTE: Partner is lost to follow-up (LTFU) if not successfully traced (definition: notified of HIV exposure and enrolled into the study) within 6 attempts; complete Partner Summary Form electronically.*  *NOTE: complete Partner Summary Form if partner is successfully traced and enrolled into the study* | | |

**APPENDIX 3: SPECIAL CIRCUMSTANCES**

1. **Invalid or missing phone or physical locator information**

**Timeline to contact index to obtain the correct contact information of partner**

| **Tracing attempt** | **Mode of Tracing** | **Timeframe for contacting index** |
| --- | --- | --- |
| Attempt 1 | Phone | On the same day partner contact information is found invalid or missing |
| Attempt 2 | Physical | Within 7 days after completion of Attempt 1; follow up to 4 weeks to obtain correct phone number or locator information |
| *NOTE: Follow Appendix 2 to trace partner if the correct phone number and locator information is obtained*  *NOTE: If index says that she doesn’t know the correct locator/phone number and the partner has been contacted up to 6 attempts by phone and is unsuccessful, consider the partner as LTFU and complete Partner Tracing Form electronically.* | | |
